# Er-Chen Decoction ameliorates metabolic dysfunction–associated steatotic liver disease via gut microbiota-barrier axis-driven hepatic metabolic reprogramming

**DOI:** 10.3389/fmicb.2026.1768664

**Published:** 2026-03-10

**Authors:** Kanlin Yang, Yueli Huang, Linze Gu, Jieyuan Li, Yongjie Ma, Pengxin Gao, Wenting Qiu, Kundong Liu, Yaxing Zhang, Haimei Liu, Jiean Xu, Jinwen Xu, Tejin Liu

**Affiliations:** 1 Research Centre of Basic Integrative Medicine, School of Basic Medical Sciences, Guangzhou University of Chinese Medicine, Guangzhou, China; 2 Department of Physiology, School of Basic Medical Sciences, Guangzhou University of Chinese Medicine, Guangzhou, China; 3 The Tenth Clinical Medical College of Guangzhou University of Traditional Chinese Medicine, Zhongshan, China; 4 Zhongshan Hospital of Traditional Chinese Medicine, Zhongshan, China

**Keywords:** aryl hydrocarbon receptor, DNMT3B, epigenetic reprogramming, Er-Chen Decoction, gut microbiota, indole derivatives, metabolic dysfunction-associated steatotic liver disease

## Abstract

**Background:**

Metabolic dysfunction-associated steatotic liver disease (MASLD) constitutes a critical global health challenge, with gut-liver axis dysfunction and metabolic endotoxemia serving as key drivers. The traditional Chinese medicinal formula Er-Chen Decoction (ECD) has proven effective in treating metabolic disorders, yet the specific mechanisms by which it modulates gut-liver crosstalk have not been fully elucidated.

**Methods:**

A mouse model of MASLD was established via a high-fat diet (HFD). The therapeutic effects of ECD were evaluated using the glucagon-like peptide-1 (GLP-1) receptor agonist semaglutide (SE) as a positive control. A comprehensive analysis of the underlying mechanisms of ECD treatment was conducted by integrating fecal metagenomic sequencing, untargeted serum metabolomic profiling, hepatic transcriptomic analysis, and molecular biology assays.

**Results:**

Treatment with ECD markedly ameliorated hepatic steatosis, insulin resistance, and hyperlipidemia, demonstrating a therapeutic efficacy comparable to that of SE. Fecal metagenomic analysis indicated that whereas SE predominantly enriched the genus *Akkermansia*, the relative abundance of *Bifidobacterium* and *Lactobacillus* was markedly and specifically elevated following ECD treatment. Serum metabolomic profiling revealed that ECD specifically activated the tryptophan-indole metabolic pathway, as evidenced by elevated concentrations of indoleacrylic acid and indole-3-acetic acid. Correlation analyses established a strong positive correlation between these indole derivatives and the bacterial genera enriched by ECD. Mechanistically, our findings suggest that elevated indoles activate the aryl hydrocarbon receptor (AHR) in the colon, upregulating tight junction proteins ZO-1 and Occludin and restoring intestinal barrier integrity, thereby significantly reducing serum lipopolysaccharide (LPS) levels. In hepatic tissue, the diminished LPS influx alleviated the suppression of DNA methyltransferase 3B (DNMT3B), thereby promoting the epigenetic silencing of the lipid droplet fusion protein CIDEA and inhibiting pathological hepatic lipogenesis.

**Conclusion:**

Our findings elucidate a novel mechanism through which ECD may ameliorate MASLD via the distinctive “gut microbiota-indole-barrier” axis. In contrast to SE, ECD modulates gut microbiota composition to boost indole production and subsequently activate AHR signaling. This activation inhibits endotoxin translocation and induces hepatic DNMT3B-mediated epigenetic reprogramming to reverse hepatic steatosis. These results offer scientific evidence supporting the potential of ECD as an effective therapeutic strategy for MASLD.

## 1 Introduction

Metabolic dysfunction-associated steatotic liver disease (MASLD), previously referred to as non-alcoholic fatty liver disease (NAFLD), is a chronic hepatic disorder characterized by excessive hepatic lipid accumulation (Feng et al., 2025). MASLD is currently recognized as the most prevalent chronic liver disease worldwide. Epidemiological data reveal that the global prevalence of MASLD is approximately 30%, with an incidence rate estimated at 46.9 cases per 1,000 person-years, and a higher prevalence observed in men (Riazi et al., 2022). The clinical spectrum of MASLD ranges from simple hepatic steatosis to metabolic dysfunction-associated steatohepatitis (MASH), which may progress to cirrhosis and hepatocellular carcinoma (Wong et al., 2023). Additionally, MASLD is closely associated with other metabolic conditions, particularly type 2 diabetes mellitus (T2DM) and obesity, creating a deleterious feedback loop that worsens patient outcomes (Cusi et al., 2025; Xiang et al., 2025), thereby imposing a substantial burden on public health.

Currently, lifestyle modification remains the cornerstone of MASLD management (European Association for the Study of the Liver et al., 2024), yet long-term adherence poses a significant challenge. Pharmacological interventions have evolved, with Resmetirom becoming the first FDA-approved therapy for MASH with liver fibrosis (Jamal et al., 2025). Other agents, such as Vitamin E, pioglitazone, and GLP-1 receptor agonists (e.g., semaglutide), are also widely employed (European Association for the Study of the Liver et al., 2024; Ore and Akinloye, 2021). However, the clinical application of these “single-target” therapies is frequently constrained by modest response rates and adverse effects. For instance, pioglitazone is linked to weight gain and fluid retention (Cusi et al., 2016), while GLP-1 receptor agonists and Resmetirom often induce gastrointestinal distress, such as nausea and diarrhea (Karagiannis et al., 2022; Shuaibi et al., 2025). Furthermore, the high cost and strict indication criteria of novel therapies may limit their widespread accessibility (Shuaibi et al., 2025). Consequently, there is a growing interest in natural products and Traditional Chinese Medicine (TCM) due to their multi-target mechanisms and favorable safety profiles (Cai et al., 2024). Recent studies have highlighted that natural active ingredients, such as Oroxin B (Huang et al., 2023), Hesperetin (Li et al., 2021), and Isoliensinine (Wang S. et al., 2025), can effectively ameliorate MASLD by modulating gut microbiota dysbiosis and regulating lipid metabolism. Similarly, extracts like *Cornus officinalis* vinegar (Cao et al., 2022) and *Potentilla anserina* polysaccharides (Lin et al., 2024) have shown promise in alleviating hepatic steatosis via the gut-liver axis.

Accumulating evidence highlights the critical role of the gut-liver axis in the pathogenesis of MASLD (Hsu and Schnabl, 2023). Gut microbiota dysbiosis compromises the integrity of the intestinal barrier, facilitating the translocation of gut-derived endotoxins, specifically lipopolysaccharides (LPS), into the portal circulation (Bian et al., 2025; Fei et al., 2020). Upon entering the liver, LPS functions as a dual-pathogenic driver. First, as a potent proinflammatory mediator, LPS activates the TLR4/ NF -κB signaling pathway, triggering the release of cytokines and driving the progression from simple steatosis to steatohepatitis (Zhou et al., 2025). Second, beyond inflammation, the LPS-TLR4 axis directly promotes hepatic steatosis by upregulating sterol regulatory element-binding protein 1c (SREBP-1c), a key transcription factor for *de novo* lipogenesis (Tian and Zhang, 2026; Zou et al., 2025). However, distinct from these well-characterized transcriptional pathways, a recent breakthrough identified LPS as a key regulator of hepatic epigenetic modification. Specifically, chronic exposure to low levels of LPS markedly downregulates the expression of DNA methyltransferase 3B (DNMT3B) in hepatocytes. The deficiency of DNMT3B leads to hypomethylation of the promoter region of cell death-inducing DFFA-like effector a (CIDEA) and its subsequent transcriptional activation, thereby directly driving hepatic lipid accumulation via an epigenetic mechanism (Li Q. et al., 2024). Therefore, regulating the gut microbiota to block the translocation of endotoxins and restore the hepatic DNMT3B-CIDEA epigenetic axis represents a promising therapeutic strategy.

Traditional Chinese Medicine (TCM) offers unique advantages in managing metabolic disorders through multi-component and multi-target interventions (Du et al., 2021; Li Y. et al., 2024; Wang M. et al., 2025). Among them, Er-Chen Decoction (ECD) stands out as a classic herbal formula with a history of over a thousand years, first documented in the Taiping Huimin Heji Ju Fang of the Song Dynasty (Shen et al., 2025). Explicitly designed for “drying dampness and resolving phlegm” (Zao-Shi-Hua-Tan), ECD is clinically regarded as a fundamental prescription for treating Phlegm-Dampness syndrome—a core pathological pattern that closely parallels the modern clinical features of obesity, hyperlipidemia, and MASLD (Lyu et al., 2025; Shen et al., 2025). In clinical practice, randomized controlled trials have demonstrated that modified ECD formulas significantly reduce body mass index (BMI) and serum lipid levels (TC and TG) in obese and type 2 diabetic populations—populations intrinsically linked to MASLD pathogenesis (Guo et al., 2025). Consistently, accumulating modern pharmacological studies have confirmed the therapeutic efficacy of ECD in alleviating hepatic steatosis and improving insulin sensitivity (Deng et al., 2024; Wang Y. et al., 2025; Zheng et al., 2025). However, the specific molecular mechanisms underlying its therapeutic effects, particularly its impact on the gut-liver epigenetic crosstalk, remain to be elucidated.

The aryl hydrocarbon receptor (AHR) is a critical sensor of gut environmental signals (Palrasu et al., 2025), and its activity is largely dependent on indole derivatives generated by tryptophan-metabolizing bacteria, such as *Lactobacillus* species (Cao et al., 2025). Activation of AHR signaling enhances intestinal barrier integrity and ameliorates liver disease (Jing et al., 2025; Wrzosek et al., 2021). Based on these findings, we hypothesized that ECD ameliorates MASLD by reshaping the gut microbiota to enrich indole-producing species, thereby activating intestinal AHR signaling to repair the mucosal barrier. This restoration effectively blocks the influx of LPS into the liver, thereby dual-targeting the pathology: eliminating the trigger for TLR4-mediated hepatic inflammation while simultaneously relieving the suppression of the hepatic DNMT3B-CIDEA axis to reduce steatosis.

In the present study, we established a high-fat diet (HFD)-induced MASLD mouse model and employed an integrated multi-omics approach, combining metagenomics, metabolomics, and transcriptomics, to systematically elucidate the therapeutic mechanisms underlying the effects of ECD. Semaglutide (SE), a GLP-1 receptor agonist with proven efficacy in resolving MASH and improving systemic metabolism (Liu et al., 2025; Newsome et al., 2021), was included as a positive control to delineate the distinct regulatory patterns of ECD. Specifically, we aimed to investigate whether ECD exerts hepatoprotective effects by modulating the “gut microbiota-indole-barrier” axis and subsequently remodeling the hepatic epigenetic landscape, thereby providing a novel scientific rationale for its clinical application.

## 2 Materials and methods

### 2.1 Preparation and quality control of ECD

The raw herbal materials comprising ECD were purchased from Kang Mei Pharmaceutical Co. Ltd. (Guangdong, China), and the detailed composition is presented in Table 1. To prepare the decoction, the mixed crude herbs were soaked in purified water at a ratio of 1:10 (w/v) for 30 min. The mixture was boiled for 30 min and subsequently maintained at a gentle simmer (approximately 100 °C) for another 30 min. After filtration, the residues were subjected to a second extraction under identical conditions. The filtrates from both extractions were combined and concentrated to a final density of 2.639 g crude drug/mL (Deng et al., 2024). The extract was prepared weekly, stored at 4 °C, and reheated to ambient temperature prior to administration. To ensure quality consistency, the chemical profile of ECD was characterized using UPLC–MS/MS analysis.

**Table 1:** Herbal composition of ECD in the experiment.

Chinese name	Botanical name	Ratio	Composition (g)
Ju Hong	*Citrus grandis* “Tomentosa”	3	15
Ban Xia	*Pinellia ternate* (Thunb.) Breit.	3	15
Fu Ling	*Poria cocos* (Schw.) Wolf	2	9
Gan Cao	*Glycyrrhiza uralensis* Fisch.	1	4.5

The chemical profile of ECD was analyzed using an Agilent 1290 Infinity II UPLC system coupled with an Agilent 6546 Quadrupole Time-of-Flight (Q-TOF) mass spectrometer (Agilent Technologies, CA, United States). Separation was achieved using an Agilent InfinityLab Poroshell 120 EC-C18 column (2.1 × 150 mm, 2.7 μm) maintained at a constant temperature of 40 °C. The mobile phase consisted of 0.1% formic acid in water (A) and (B) acetonitrile. The gradient elution program was set as follows: 0–2 min, 5% B; 2–8 min, 5–50% B; 8–25 min, 50–95% B; 25–30 min, 95% B; 30–30.1 min, 95–5% B; and 30.1–35 min, 5% B for equilibration. The flow rate was maintained at 0.2 mL/min. Mass spectrometric detection was conducted utilizing a Dual Agilent Jet Stream (AJS) ESI source operating in Auto MS/MS mode. The key source parameters were as follows: gas temperature, 320 °C; drying gas flow, 8 L/min; nebulizer pressure, 35 psig; sheath gas temperature, 350 °C; sheath gas flow, 11 L/min; capillary voltage, 3,500 V; nozzle voltage, 1,000 V; fragmentor voltage, 175 V; and skimmer, 65 V. Data were acquired in the mass range of 100–1,100 m/z. Compound identification was performed using the Agilent MassHunter Workstation Software by matching the spectra against the Agilent Natural Standard NPs PCDL with a mass error tolerance of 20 ppm.

### 2.2 Animal models and treatment

The study utilized male C57BL/6J mice (6–8 weeks old) sourced from Guangdong Medical Laboratory Animal Center (Guangzhou, China; License No. SCXK (Yue) 2022-0002). All animal experiments were approved by the Animal Ethics Committee of Guangzhou University of Chinese Medicine (Approval No.20240612003) and were conducted in strict accordance with the National Institutes of Health Guide for the Care and Use of Laboratory Animals. The animals were housed in a specific-pathogen-free (SPF) facility at the Research Center of Basic Integrated Medicine, Guangzhou University of Chinese Medicine (License No. SYXK [Yue] 2023-0182), under controlled environmental conditions (22 ± 2 °C; 12-h light/dark cycle) with *ad libitum* access to food and water.

Following a one-week acclimatization period, the mice were randomly allocated into four groups (*n* = 9 per group) based on body weight to ensure uniformity in baseline conditions: Control, Model, Er-Chen Decoction (ECD), and semaglutide (SE). The Control group was fed a standard chow diet, whereas the Model, ECD, and SE groups were fed a high-fat diet (HFD, 60% energy from fat, 5.24 kcal/g; Cat. No. GD60, Guangdong Medical Laboratory Animal Center) formulated based on Research Diets D12492 to induce MASLD. HFD feeding lasted for 12 weeks to establish the MASLD model (Zhao et al., 2025). From weeks 13 to 20, the mice received specific treatments while maintaining their respective diets. ECD via intragastric gavage at a daily dose of 26.39 g crude drug/kg (calculated based on the weight of raw herbal materials). This dosage corresponds to 4 times the clinical human equivalent dose, calculated based on the body surface area (BSA) normalization method (human-to-mouse conversion factor of 9.1) (Deng et al., 2024). The SE group received a daily subcutaneous injection of semaglutide (Novo Nordisk A/S, Denmark) at a dose of 30 nmol/kg/day (Yue et al., 2022). The Control and Model groups were administered an equivalent volume (10 mL/kg) of saline intragastrically.

Weekly body weight was recorded. An oral glucose tolerance test (OGTT) was performed in the 19th week. At the end of the 20-week experimental period, mice were euthanized for serum and tissue collection. The terminal procedure was conducted under deep anesthesia induced by inhalation of 3–4% isoflurane. Upon reaching a surgical plane of anesthesia (loss of righting reflex and absence of response to toe pinch), blood was collected from the retro-orbital sinus using a capillary tube. Subsequently, euthanasia was completed by cervical dislocation, which was performed only by personnel who had demonstrated proficiency in the technique. Death was confirmed by the absence of a heartbeat, respiration, and corneal reflex. Following this, liver and colon tissues were rapidly dissected, weighed, and either snap-frozen in liquid nitrogen or fixed in 4% paraformaldehyde for subsequent analyses.

### 2.3 Histological analysis

Fresh liver and colon tissues were fixed in 4% paraformaldehyde for 24 h, dehydrated, embedded in paraffin, and sectioned into 4-μm sections. The sections were stained using a Hematoxylin and Eosin (H&E) Staining Kit (Cat. No. C0105M, Beyotime, Shanghai, China) to assess general tissue morphology and inflammatory infiltration. For the evaluation of hepatic lipid accumulation, fresh liver tissues were embedded in OCT compound and cryosectioned at 8 μm thickness using a cryostat. The frozen sections were then stained with Oil Red O working solution (Cat. No. O0625, Sigma-Aldrich, MO, United States) for 20 min at room temperature in the dark, followed by differentiation in 60% isopropanol and counterstaining with hematoxylin. Representative images of each group were acquired using a light microscope (Olympus BX46F, Olympus, Tokyo, Japan). Scale bars (200 μm for colon tissues and 50 μm for liver tissues) were integrated into the images for dimensional reference.

### 2.4 Biochemical analysis

At week 19, an oral glucose tolerance test (OGTT) was performed following a 6-h fast with ad libitum access to water. Mice were administered glucose (2.0 g/kg) via oral gavage, and blood glucose levels were measured in tail vein blood at 0, 30, 60, 90, and 120 min post-administration using an Accu-Chek Active meter (Roche Diagnostics, Germany).

At the experimental endpoint, blood samples were collected and centrifuged (3,500 rpm, 10 min, 4 °C) to obtain serum. Serum levels of alanine aminotransferase (ALT), aspartate aminotransferase (AST), total cholesterol (TC), and triglycerides (TG) were determined using a Mindray BS-460 Automatic Biochemical Analyzer (Shenzhen Mindray Bio-Medical Electronics Co., Ltd., Shenzhen, China) according to the manufacturer’s instructions. Hepatic TG concentrations were quantified using a commercial assay kit (Cat. No. A110-1-1, Nanjing Jiancheng Bioengineering Institute, Nanjing, China) following the standard protocol.

### 2.5 Metagenomic sequencing and analysis

Fresh fecal samples were collected, snap-frozen in liquid nitrogen, and stored at −80 °C. Genomic DNA was extracted from approximately 0.2 g of fecal samples utilizing the FastPure Stool DNA Isolation Kit (Magnetic Bead) (MJYH, Shanghai, China), adhering strictly to the manufacturer’s protocol. DNA concentration and purity were assessed using a TBS-380 fluorometer and NanoDrop 2000 spectrophotometer, respectively, while DNA integrity was confirmed via 1% agarose gel electrophoresis. For library construction, genomic DNA was fragmented to an average size of approximately 350 bp using a Covaris M220 instrument (Gene Company Limited, China). Sequencing libraries were prepared using the NEXTFLEX® Rapid DNA-Seq Kit (Bioo Scientific, Austin, TX, United States). Subsequently, the libraries were sequenced on the Illumina NovaSeq X Plus platform (Illumina, San Diego, CA, United States) at Majorbio Bio-Pharm Technology Co., Ltd. (Shanghai, China) to generate paired-end reads.

The raw sequencing data were quality-filtered using fastp (Chen et al., 2018) to remove adapters and low-quality reads. To remove host contamination, clean reads were aligned to the *Mus musculus* genome (Vertebrates: mus_musculus_c57bl6nj) using BWA (Li and Durbin, 2009). The remaining high-quality reads were assembled into contigs utilizing MEGAHIT (Li et al., 2015). Contigs with a length of ≥ 300 base pairs were selected as the final assembly result. Subsequently, open reading frames (ORFs) were predicted from each assembled contig using Prodigal (Hyatt et al., 2010), and ORFs with a length ≥ 100 bp were retrieved. Gene abundance was calculated using SOAPaligner (Li et al., 2008). Taxonomic annotation was performed by aligning the non-redundant gene catalog against the NCBI Non-Redundant (NR) database using DIAMOND (Buchfink et al., 2015) with an *e*-value threshold of 1 × 10^−5^. The relative abundance at each taxonomic level was determined based on gene abundance. Differentially abundant bacterial taxa among the groups were determined using Analysis Effect Size (LEfSe) analysis, with a significance threshold set at an LDA score of > 3.0. All bioinformatics analyses were conducted on the Majorbio Cloud Platform^1^ (Ren et al., 2022).

### 2.6 Untargeted metabolomics analysis

Serum metabolites were extracted using a mixture of methanol and acetonitrile (1:1, v/v) spiked with internal standards. Following sonication, protein precipitation was carried out at −20 °C, followed by centrifugation. The resulting supernatants were evaporated to dryness and reconstituted in acetonitrile:water (1:1, v/v) for analysis. LC–MS/MS analysis was conducted using a Thermo Scientific Vanquish UHPLC system coupled to an Orbitrap Exploris 240 mass spectrometer. Chromatographic separation was performed on an ACQUITY UPLC HSS T3 column (100 mm × 2.1 mm, 1.8 μm; Waters, Milford, MA, United States) maintained at 40 °C. The mobile phases consisted of 95% water and 5% acetonitrile containing 0.1% formic acid (A) and 47.5% acetonitrile, 47.5% isopropanol, and 5% water containing 0.1% formic acid (B). The injection volume was set at 3 μL. Raw data processing, including peak alignment and identification against the HMDB and METLIN databases, was performed using Progenesis QI software (Waters). Differential metabolites were identified on the Majorbio Cloud Platform based on the criteria of Variable Importance in Projection (VIP) > 1 and *p* < 0.05.

### 2.7 Western blot and enzyme-linked immunosorbent assay (ELISA) analysis

Colon and liver tissues were accurately weighed and homogenized in ice-cold RIPA Lysis Buffer (Medium) (Cat. No. FD008, Fude Biological Technology, Hangzhou, China) at a ratio of 1:10 (w/v, 30 mg tissue in 300 μL buffer). The buffer was supplemented with protease inhibitor (Cat. No. FD1001) and phosphatase inhibitor (Cat. No. FD1002) cocktails at a 1:100 dilution. The lysates were centrifuged at 12,000 × g for 15 min at 4 °C to collect the supernatant. Protein concentration was determined using a BCA Protein Assay Kit. Equal amounts of protein (30 μg) were separated by 10% SDS-PAGE and electrotransferred onto 0.22 μm PVDF membranes (Millipore, MA, United States). The membranes were blocked with Fast Blocking Buffer (Cat. No. G2052, Servicebio, Wuhan, China) at room temperature for 15 min and subsequently incubated overnight at 4 °C with primary antibodies.

The primary antibodies used in this study included: anti-AHR (1:1000, Cat. No. 67785-1-lg, Proteintech, Wuhan, China), anti-Occludin (1:1000, Cat. No. GB111401-50, Servicebio, Wuhan, China), anti-TNF-α (1:5000, Cat. No. YM8306, ImmunoWay, TX, United States), anti-IL-1β (1:1000, Cat. No. 12242S, Cell Signaling Technology, MA, United States), anti-PPARγ (1:5000, Cat. No. YM8211, ImmunoWay), anti-CIDEA (1:1000, Cat. No. A7655, ABclonal, Wuhan, China), and anti-DNMT3B (1:1000, Cat. No. A11079, ABclonal). Anti-GAPDH (1:5000, Cat. No. AF7021, Affinity Biosciences, Jiangsu, China) and anti-β-actin (1:20000, Cat. No. 66009-1-Ig, Proteintech) were used as loading controls.

After three washing steps with TBST (10 min each), the membranes were incubated with HRP-conjugated anti-rabbit (Cat. No. 7074S) or anti-mouse (Cat. No. 7076S) secondary antibodies (1:2000, Cell Signaling Technology) for 1.5 h at room temperature. Following three additional washing steps with TBST, protein bands were visualized using an ECL chemiluminescence kit and imaged on a Tanon 5200ce Chemiluminescence Imaging System. Band intensity was quantified using ImageJ software (NIH, Bethesda, MD, United States) and normalized to GAPDH or β-actin levels.

For endotoxin analysis, serum LPS levels were measured using a Mouse Lipopolysaccharides ELISA Kit (Cat. No. JL20691, Shanghai Jianglai Biotechnology Co., Ltd., Shanghai, China) strictly according to the manufacturer’s protocol. Absorbance was measured at 450 nm using a microplate reader.

### 2.8 Immunofluorescence staining

Paraffin-embedded colon tissues were sectioned at 4 μm thickness, deparaffinized in xylene, and rehydrated through a graded ethanol series. Antigen retrieval was performed by 95 °C heating in TRIS-EDTA Antigen Retrieval Solution (Cat. No. BL617A, Biosharp, Hefei, China) for 15 min to expose the specific binding sites. After cooling to room temperature and washing with PBS, the sections were blocked with 10% Goat serum for 1 h at room temperature to prevent non-specific binding. Subsequently, the sections were incubated overnight at 4 °C with the primary antibody against ZO-1 (1:200, Cat. No. YM8448, ImmunoWay, TX, United States).

Following three washes with PBS, the sections were incubated with Alexa Fluor 594-conjugated goat anti-rabbit IgG (1:1000, Cat. No. A11012, Thermo Fisher Scientific, MA, United States) for 1 h at room temperature in the dark. Finally, the sections were mounted and nuclei counterstained using Antifade Mounting Medium with DAPI (Cat. No. P0131, Beyotime, Shanghai, China). Fluorescence images were acquired using a Zeiss LSM800 confocal laser scanning microscope (Carl Zeiss, Oberkochen, Germany). The acquisition parameters were standardized across all samples to ensure comparable signal intensity.

### 2.9 Transcriptomic analysis

Total RNA was isolated from liver tissues utilizing the SteadyPure Universal RNA Extraction Kit (Accurate Biology, Hunan, China), adhering strictly to the manufacturer’s protocol. RNA quality and integrity were determined by 1% agarose gel electrophoresis and the Agilent 5300 Fragment Analyzer (Agilent Technologies, CA, United States). Only high-quality RNA samples (total amount ≥ 0.5 μg, concentration ≥ 20 ng/μL, RQN > 4.5) were used for the library construction. Sequencing libraries were prepared utilizing the Illumina® Stranded mRNA Prep, Ligation Kit (Illumina, San Diego, CA, United States) in accordance with the manufacturer’s protocol. The libraries were then sequenced on the Illumina NovaSeq X Plus platform (Illumina, San Diego, CA, United States) to generate paired-end reads. Raw reads were filtered using fastp to remove adapters and low-quality reads (Chen et al., 2018). The clean reads were aligned to the *Mus musculus* reference genome (GRCm39) using HISAT2 (Kim et al., 2015). Gene expression levels were quantified using RSEM software (Li and Dewey, 2011), and the expression abundance was normalized to Transcripts Per Million (TPM). Differential expression analysis between groups was performed using DESeq2 (Love et al., 2014). Differentially expressed genes (DEGs) were identified using the criteria of a false discovery rate (FDR) less than 0.05 and an absolute log2 fold change (|log_2_FC|) of at least 1.

### 2.10 Quantitative real-time PCR (RT-qPCR)

Total RNA was isolated from liver and colon tissues utilizing the SteadyPure Universal RNA Extraction Kit (Accurate Biology, Hunan, China), adhering strictly to the manufacturer’s protocol. Briefly, approximately 20–30 mg of fresh tissue was homogenized in 600 μL of lysis buffer provided in the kit. RNA concentration and purity were measured using a NanoDrop One spectrophotometer (Thermo Fisher Scientific, WI, United States). Subsequently, 1 μg of total RNA was reverse-transcribed into cDNA using the 4× EZscript Reverse Transcription Mix II (with gDNA Remover) (Cat. No. EZB-RT2GQ, EZBioscience, Roseville, MN, United States). RT-qPCR was conducted on an Applied Biosystems QuantStudio 5 Real-Time PCR System using the 2× Color SYBR Green qPCR Master Mix (ROX2) (Cat. No. A0012-R2, EZBioscience). The cycling conditions were as follows: initial denaturation at 95 °C for 5 min, followed by 40 cycles of 95 °C for 10 s and 60 °C for 30 s. Melting curve analysis was performed to verify the specificity of PCR products. The primers used for the target genes (*Dnmt3b*, *Cidec*, *Cidea*, *Pparg*, *Tnf*, *Il1b, Cyp1a1, Cyp1b1*) and internal controls (*Actb* and *18S*) are listed in Supplementary Table S1. The comparative 2^ ^-ΔΔCt^ was utilized to analyze relative mRNA abundance, with normalization to the geometric mean of the reference genes.

### 2.11 Statistical analysis

GraphPad Prism 10.0 (GraphPad Software, San Diego, CA, United States) was used for both statistical evaluation and graphical representation. All data are expressed as the mean ± standard deviation (SD). Data normality was verified using the Shapiro–Wilk test, and differences among groups were analyzed using One-way Analysis of Variance (ANOVA) followed by Dunnett’s multiple comparisons test to compare the treatment groups against the Model group. Bioinformatics analyses were performed utilizing the Majorbio Cloud Platform (see Footnote 1). Beta-diversity and metabolic profile differences were assessed using ANOSIM, PCA, and PLS-DA. Differentially abundant taxa were identified by LEfSe (LDA > 3.0), differential metabolites were screened based on VIP > 1 and *p* < 0.05, and differentially expressed genes were identified using DESeq2 (adjusted *p* < 0.05). For the comparison of specific omics features (e.g., bacterial abundance and metabolite intensity), the non-parametric Kruskal-Wallis H test was used, followed by Tukey–Kramer post-hoc tests with False Discovery Rate (FDR) correction. Correlations were evaluated using Spearman’s rank analysis. All results were considered statistically significant at *p* < 0.05.

## 3 Results

### 3.1 LC–MS/MS-based chemical profiling of water extracts of ECD

**Figure 1:**
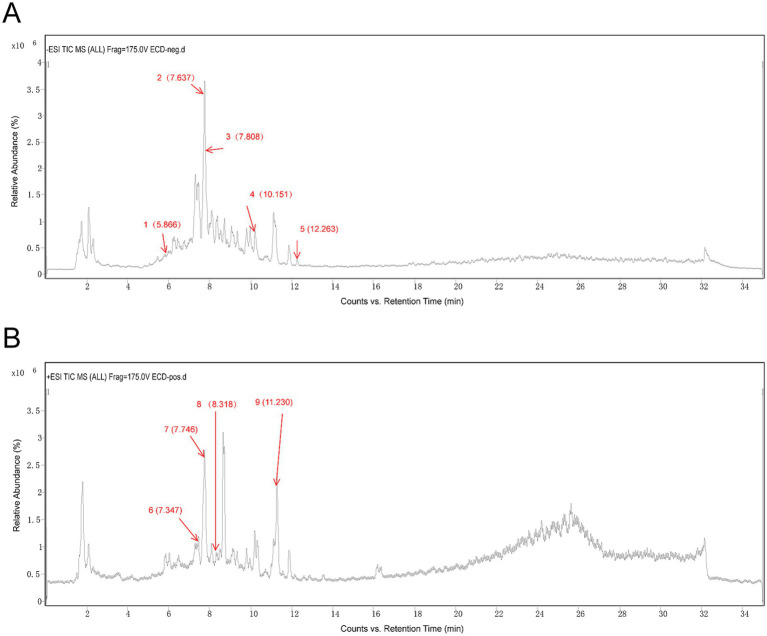
Chemical profile of Er-Chen Decoction (ECD). Total ion chromatograms (TIC) of the ECD extract analyzed by UPLC-MS/MS. **(A)** Total ion chromatogram (TIC) in negative ion mode. **(B)** Total ion chromatogram (TIC) in positive ion mode. The arrows indicate the retention times of the nine representative chemical constituents identified in this study. Detailed information for these compounds is listed in Table 2.

To elucidate the chemical components of ECD, the chemical fingerprint of the ECD extract was comprehensively characterized using UPLC-MS/MS. The total ion chromatograms (TIC) revealed a complex chemical profile (Figure 1). By matching the accurate mass and MS/MS fragmentation patterns against the Agilent Natural Standard NPs PCDL database, we screened and tentatively identified a series of constituents. The complete list of compounds identified in this study is provided in Supplementary Table S2. Based on this profile, nine representative compounds with potential biological significance were selected for further investigation (Table 2). The retention times of these key constituents are indicated by arrows in the TIC (Figure 1), and their corresponding extracted ion chromatograms (EIC) and mass spectra are presented in Supplementary Figure S1. These compounds included L-tryptophan, hesperetin 7-O-glucoside, naringin, glycyrrhizic acid, 6-gingerol, narirutin, aromadendrin, liquiritin, and limonin. Notably, the presence of L-tryptophan, a biosynthetic precursor of indole derivatives, together with anti-inflammatory agents such as naringin and glycyrrhizic acid, suggests that ECD may provide potential substrates for the tryptophan-indole metabolic pathway, while collectively contributing to the regulation of hepatic inflammatory processes.

**Table 2:** Identification of major compounds in ECD by UPLC-MS/MS.

No.	Component name	Formula	RT (min)	m/z	Mass error (ppm)	Ion form
1	L-Tryptophan	C_11_H_12_N_2_O_2_	5.896	203.0825	−0.5	[M-H]^−^
2	Hesperetin 7-O-glucoside	C_22_H_24_O_11_	7.679	463.1272	5.65	[M-H]^−^
3	Naringin	C_27_H_32_O_14_	7.768	579.1747	4.78	[M-H]^−^
4	Glycyrrhizic acid	C_42_H_62_O_16_	10.168	821.3975	1.21	[M-H]^−^
5	6-Gingerol	C_17_H_26_O_4_	12.264	293.176	0.57	[M-H]^−^
6	Narirutin	C_27_H_32_O_14_	6.977	581.1857	−1.35	[M+H]^+^
7	Aromadendrin	C_15_H_12_O_6_	7.332	289.0706	−0.22	[M+H]^+^
8	Liquiritin	C_21_H_22_O_9_	7.348	419.1336	−0.14	[M+H]^+^
9	Limonin	C_26_H_30_O_8_	11.219	471.2016	0.54	[M+H]^+^

### 3.2 ECD alleviated systemic metabolic disorders and hepatic steatosis in MASLD mice

**Figure 2:**
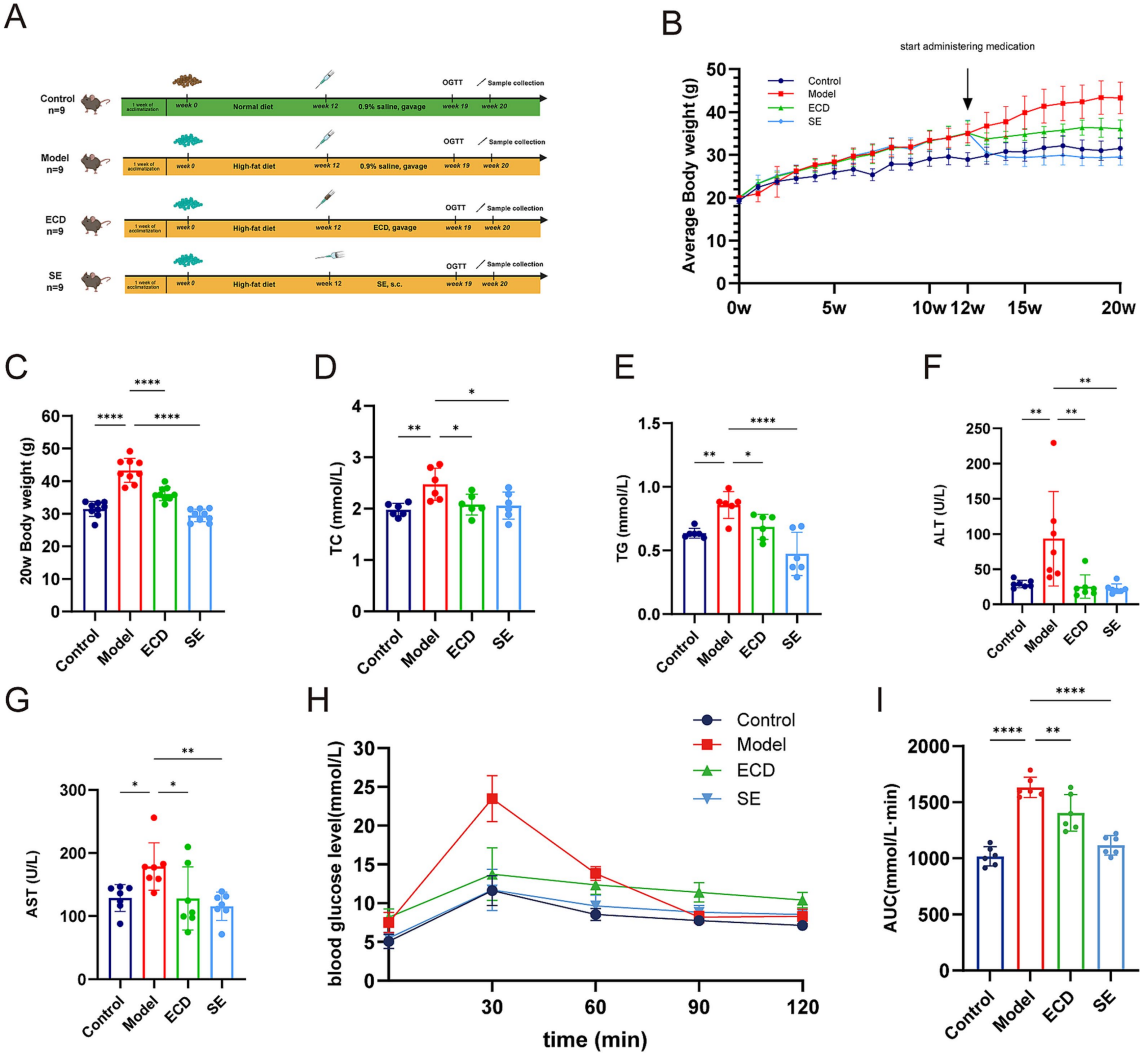
ECD alleviates HFD-induced systemic metabolic dysregulation in MASLD mice. **(A)** Schematic diagram of the experimental design. Created with bioRender.com. **(B)** Body weight trajectory over the 20 week period. **(C)** Body weight at the experimental endpoint (week 20). **(D–G)** Serum levels of total cholesterol (TC), triglyceride (TG), alanine aminotransferase (ALT), and aspartate aminotransferase (AST). **(H)** Blood glucose levels during the oral glucose tolerance test (OGTT). **(I)** Area under the curve (AUC) for OGTT. Data are presented as mean ± SD (*n* = 6–9). Significance is denoted as *p < 0.05, **p < 0.01, ***p<0.001, ****p < 0.0001, and ns, not significant.

To assess the therapeutic efficacy of ECD, we developed a MASLD mouse model utilizing an HFD. The experimental design was shown in Figure 2A. The successful induction of obesity by HFD was confirmed by the significant increase in body weight observed in the Model group compared to the Control group (Figure 2B). However, ECD treatment effectively suppressed body weight gain throughout the experiment and significantly reduced the final body weight at week 20 (Figure 2C). Systemic metabolic disorders were also assessed. The Model group presented with severe dyslipidemia and liver injury, characterized by markedly elevated serum TG, TC, ALT, and AST levels. ECD intervention significantly reversed these pathological changes (Figures 2D–G). Furthermore, glucose metabolism was assessed through the administration of an oral glucose tolerance test (OGTT). The Model group showed impaired glucose tolerance, whereas ECD treatment significantly lowered blood glucose levels at various time points (Figure 2H) and reduced the area under the curve (AUC) (Figure 2I), indicating an improvement in systemic insulin sensitivity.

**Figure 3:**
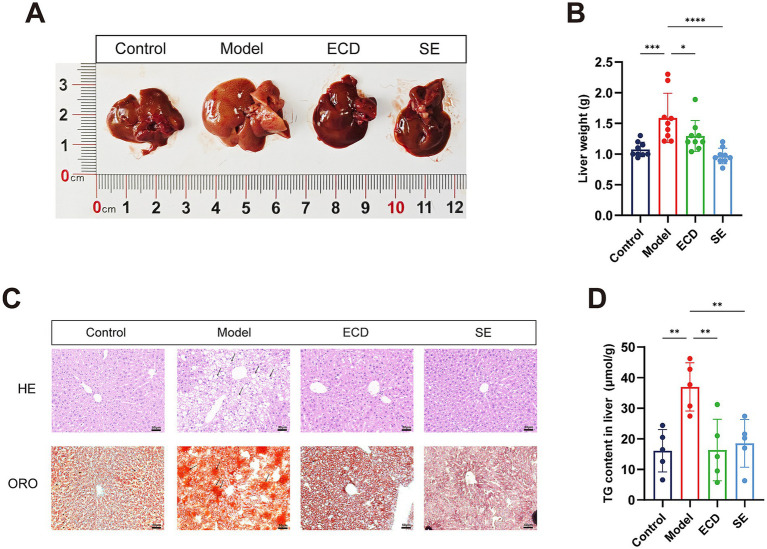
ECD alleviated hepatic steatosis and injury in HFD-induced MASLD mice. **(A)** Representative gross appearance of the livers in each group. **(B)** Liver weight at 20 weeks (*n* = 9). **(C)** Representative histological images of liver tissues stained with H&E and Oil Red O (scale bar: 50 μm). Black arrows in the H&E images indicate characteristic macrovesicular steatosis and hepatocyte ballooning in the Model group. In the Oil Red O images, black arrows highlight areas of extensive intracellular lipid droplet accumulation. **(D)** Quantitative analysis of hepatic triglyceride (TG) content (*n* = 5). Data are presented as mean ± SD. Significance is denoted as *p < 0.05, **p < 0.01, ***p < 0.001, ****p < 0.0001, and ns, not significant.

We further examined the protective effects of ECD on liver pathologies. Gross examination showed that the livers from the Model group were enlarged and pale yellow, typical of fatty liver, whereas ECD treatment restored a reddish-brown appearance similar to that of the Control group (Figure 3A). Compared to the Model group, which exhibited a substantial increase in liver mass at the 20-week endpoint, ECD administration resulted in a notable reduction in liver weight (Figure 3B). Histologically, H&E staining revealed severe pathological alterations in the Model group compared to the Control group. The liver tissue exhibited diffuse macrovesicular steatosis (characterized by large cytoplasmic vacuoles displacing the nucleus, indicated by black arrows) and widespread hepatocyte ballooning (swollen cells with rarefied cytoplasm). Additionally, the normal radial arrangement of hepatic cords was disordered, accompanied by scattered inflammatory cell infiltration. Consistent with these findings, Oil Red O staining visualized substantial intracellular lipid droplet accumulation (stained brilliant red) in the Model group. In contrast, ECD treatment markedly attenuated these pathological changes, restoring the hepatic architecture and significantly decreasing both the quantity and size of lipid droplets (Figure 3C). Consistent with the histological findings, quantitative analysis confirmed that the hepatic TG content was significantly reduced in the ECD group compared to that in the Model group (Figure 3D).

### 3.3 ECD ameliorates HFD-induced gut microbiota dysbiosis

**Figure 4:**
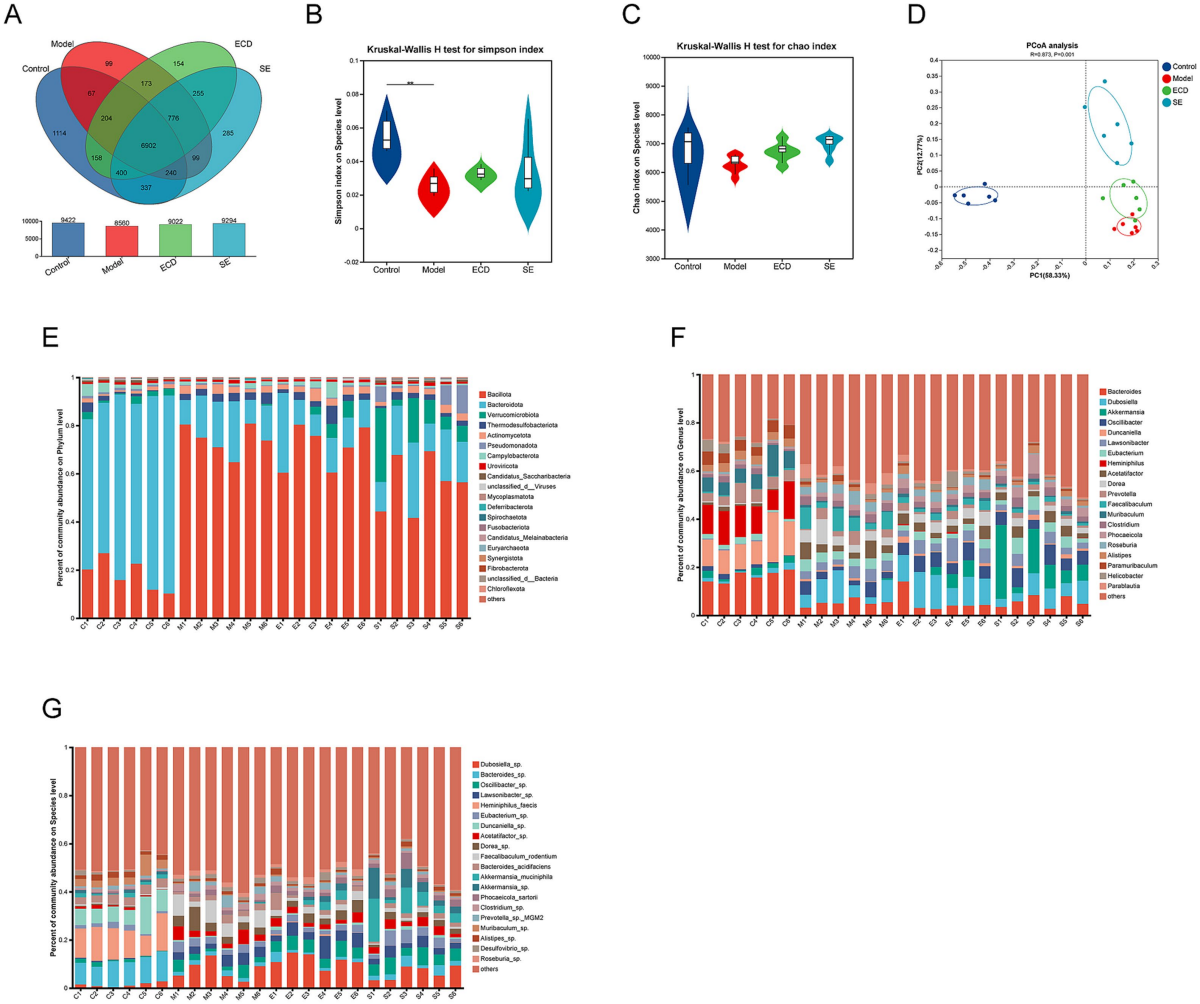
ECD alleviates the gut microbiota dysbiosis induced by HFD. **(A)** Venn diagram showing the common and unique species among the four groups. **(B,C)** α-diversity indices, including Simpson **(B)** and Chao1 **(C)**. **(D)** Principal coordinates analysis (PCoA) of β-diversity based on Bray-Curtis distance. **(E–G)** Stacked bar plots of relative microbial abundance showing the composition of the top 20 most abundant taxa at the phylum **(E)**, genus **(F)**, and species **(G)** levels. Each bar represents an individual sample. Data are presented as mean ± SD (*n* = 6). Significance is denoted as **p < 0.01.

To investigate the regulatory effect of ECD on the gut microbiome, we performed a metagenomic sequencing analysis. Venn diagram analysis revealed the extent of species intersection among the groups, reflecting specific alterations in the microbial repertoire (Figure 4A). We first examined the α-diversity to evaluate microbial richness and evenness. HFD feeding significantly decreased the Chao1 and Simpson indices compared with the Control group. However, ECD treatment ameliorated this trend, showing a tendency to improve microbial diversity compared to the Model group (Figures 4B,C). We then assessed the overall microbial community structure using β-diversity analyses. As shown in the PCoA plot (Figure 4D), a distinct separation was observed between the Model and Control groups (R=0.272, *p*=0.001), confirming the profound impact of HFD on the gut microbiome. Importantly, the overall microbial composition of ECD-treated mice was clearly separated from that of the Model group, showing a trend of recovery toward the Control phenotype. Further examination at the phylum (Figure 4E), genus (Figure 4F), and species (Figure 4G) levels confirmed that ECD modulated the relative abundance of dominant taxa, effectively correcting the HFD-induced deviations. Collectively, these findings indicate that ECD effectively ameliorates HFD-induced gut microbiota dysbiosis.

### 3.4 ECD exerts unique regulatory effects on key gut microbiota compared to SE

**Figure 5:**
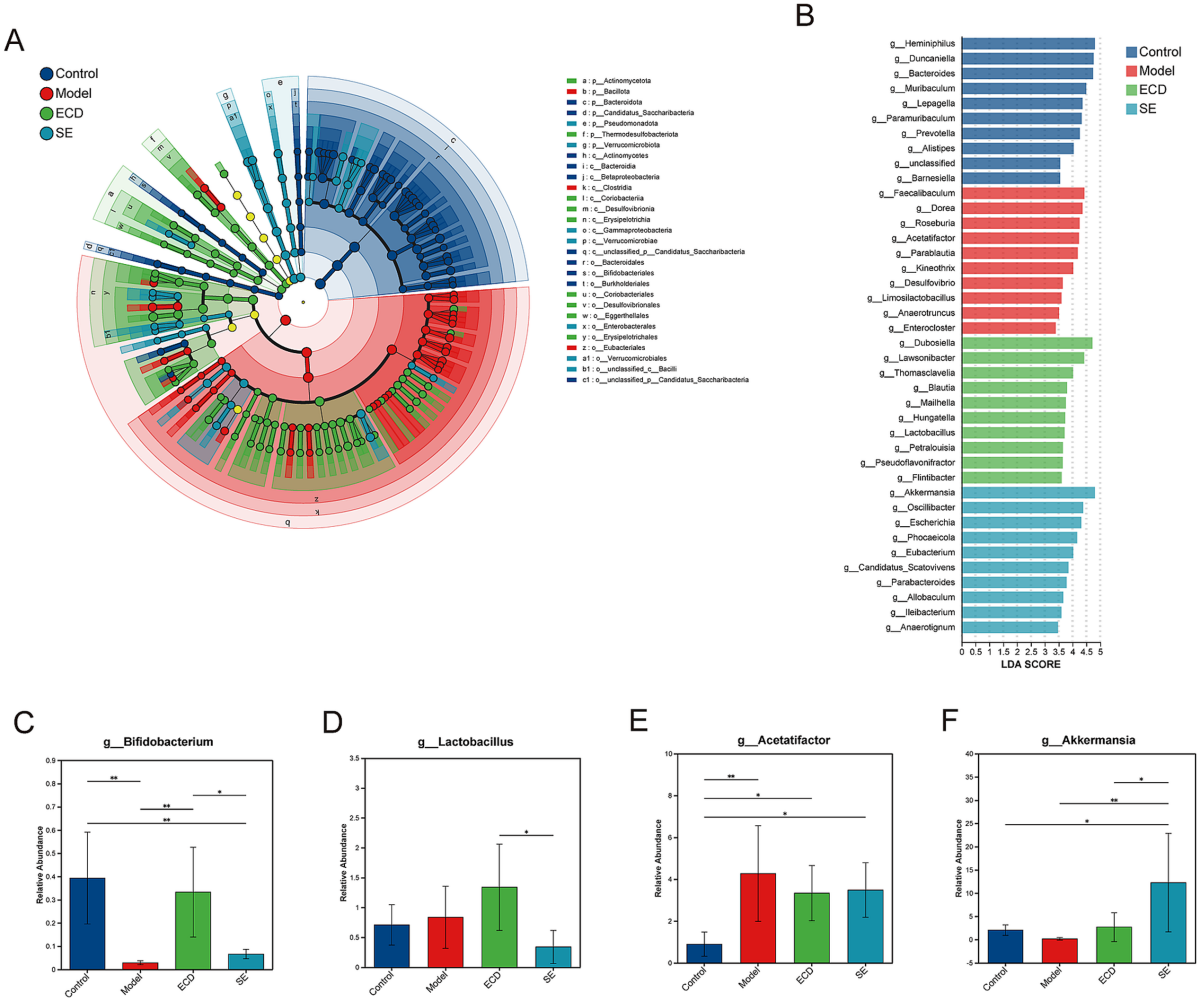
ECD and SE differentially modulate key microbial biomarkers in MASLD. **(A)** LEfSe cladogram illustrating all differential taxa (LDA score > 3.0). **(B)** Bar plot of LDA scores for differential taxa at the genus level. **(C–F)** Relative abundance of key differential genera: *Bifidobacterium* **(C)**, *Lactobacillus* **(D)**, *Acetatifactor* **(E)**, and *Akkermansia* **(F)**. Data are expressed as mean ± SD (*n* = 6/group). Significance is denoted as *p < 0.05, **p < 0.01.

To identify specific bacterial taxa driving the structural changes, we performed LEfSe analysis (Figure 5A). As shown in the LDA score plot (Figure 5B), specific biomarkers were identified for each experimental group. SE treatment significantly increased the abundance of *Akkermansia* (Figure 5F). ECD treatment resulted in a distinct modulation pattern. In contrast to the SE, the ECD did not significantly alter *the Akkermansia* levels. In contrast, ECD uniquely and significantly enriched the abundance of *Bifidobacterium* (Figure 5C) and *Lactobacillus* (Figure 5D) in the gut. Additionally, both ECD and SE treatments exhibited a trend of suppressed HFD-induced opportunistic pathogens, such as *Acetatifactor* (Figure 5E). These results indicate that while both ECD and SE improve MASLD, ECD operates through a distinct microbiome-modulating mechanism characterized by specific enrichment of *Lactobacillus* and *Bifidobacterium.* Given that these genera are known to participate in tryptophan metabolism, their enrichment suggests that ECD may exert its hepatoprotective effects by modulating downstream tryptophan metabolites.

### 3.5 ECD activates tryptophan-indole metabolism and increases serum indole derivatives

**Figure 6:**
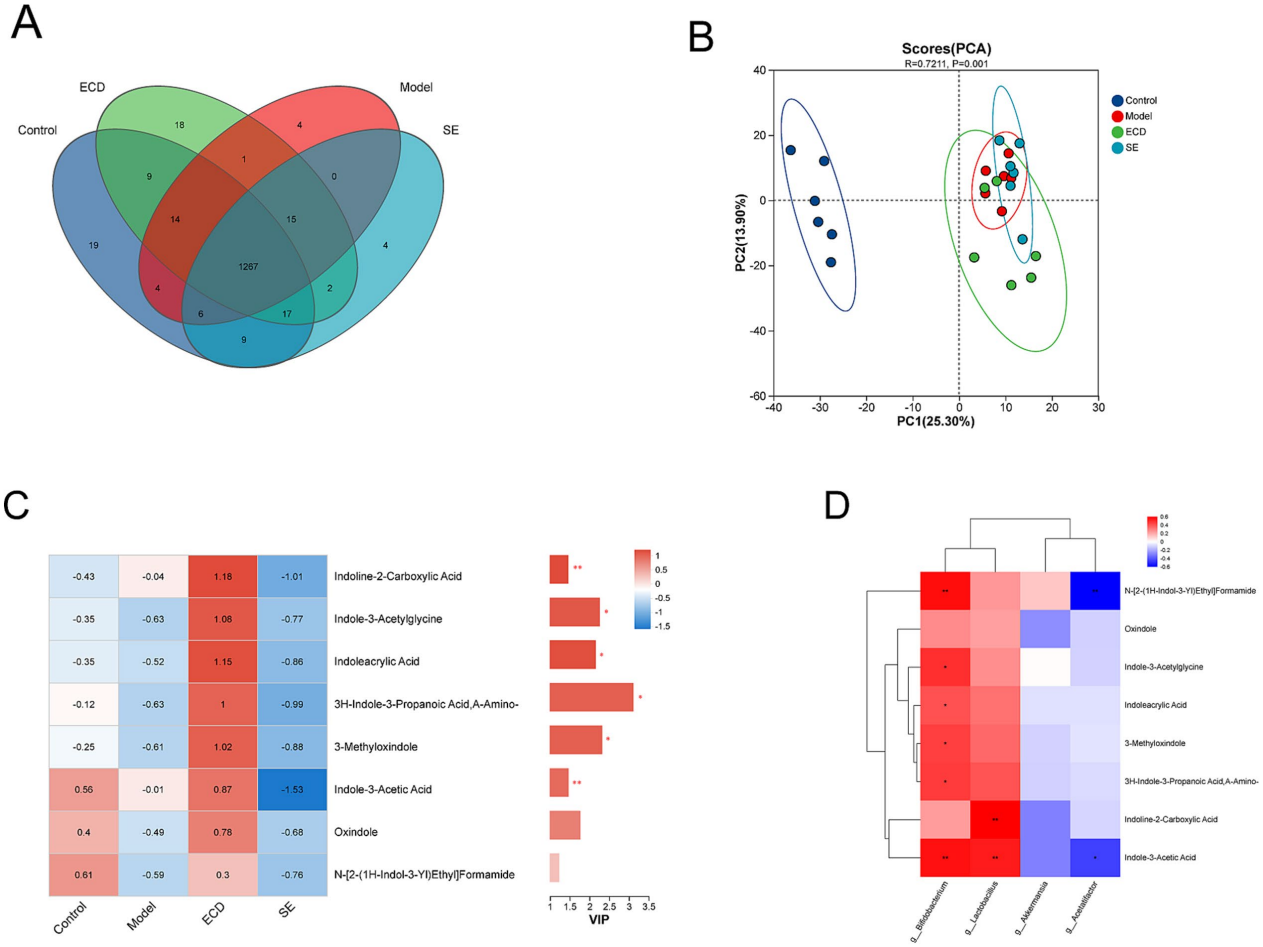
ECD modulates serum metabolites and reveals a correlation between key microbiota and indole derivatives. **(A)** Venn diagram showing the common and unique metabolites among the four groups. **(B)** PCA scores plot of serum metabolomics (*R* = 0.7211, *p* = 0.001). **(C)** Relative abundance heatmap (left) and VIP/*p*-value plot (right) of indole-related metabolites. **(D)** Spearman correlation heatmap between the key genera shown in Figure 5 and the key indole-related metabolites shown in **(C)**. Data are expressed as mean ± SD (*n* = 6 / group). In the heatmaps **(C,D)**, red indicates high abundance/positive correlation, and blue indicates low abundance/negative correlation. Significance is denoted as *p < 0.05, **p < 0.01.

To systematically evaluate the metabolic impact of ECD, we performed an untargeted serum metabolomics analysis. The distribution of common and distinct metabolites among the four groups is shown in the Venn diagram (Figure 6A). Principal Component Analysis (PCA) revealed a clear separation in the metabolic profiles between the Control and Model groups, indicating significant metabolic dysregulation in MASLD. Notably, the metabolic profile of the ECD group shifted distinctly away from the Model group and clustered toward the Control group (*R* = 0.7211, *p* = 0.001), indicating a global reversal of HFD-induced metabolic deviations (Figure 6B). A detailed list of these significantly different metabolites is provided in Supplementary Table S3. Based on our metagenomic findings that ECD specifically enriched tryptophan-metabolizing bacteria (*Lactobacillus* and *Bifidobacterium*), we focused our analysis on tryptophan-derived metabolites. Heatmap and VIP analysis revealed that the levels of indole derivatives were generally suppressed in the Model group. However, ECD treatment uniquely and significantly upregulated a cluster of key metabolites in this pathway, including 3-methyloxindole, indole-3-acetylglycine, indoleacrylic acid, and indole-3-acetic acid (Figure 6C). In contrast, the SE group did not exhibit similar activation.

To further verify whether this metabolic shift was driven by the altered gut microbiota, we performed a Spearman correlation analysis (Figure 6D). Notably, the levels of key indole derivatives were positively correlated with the abundance of *Lactobacillus* and *Bifidobacterium* (*p* < 0.05). In contrast, the opportunistic pathogen *Acetatifactor* exhibited an inverse relationship with these metabolites. These statistical associations support the notion that ECD-induced gut microbiota remodeling, particularly the enrichment of indole-producing bacteria, is a key factor in the activation of the tryptophan-indole metabolic axis. Furthermore, we evaluated the associations between the altered gut microbiota/metabolites and host lipid phenotypes. As shown in Supplementary Figure S2, key indole derivatives exhibited negative correlations with serum TG and TC levels, supporting a potential link between these metabolic changes and lipid regulation.

### 3.6 ECD promotes barrier repair and reduces endotoxemia by activating the microbiota-indole-AHR axis

Given that the indole derivatives, such as indole-3-acetic acid, identified above function as natural ligands for the aryl hydrocarbon receptor (AHR), we examined whether their systemic accumulation leads to the activation of the intestinal AHR pathway.

**Figure 7:**
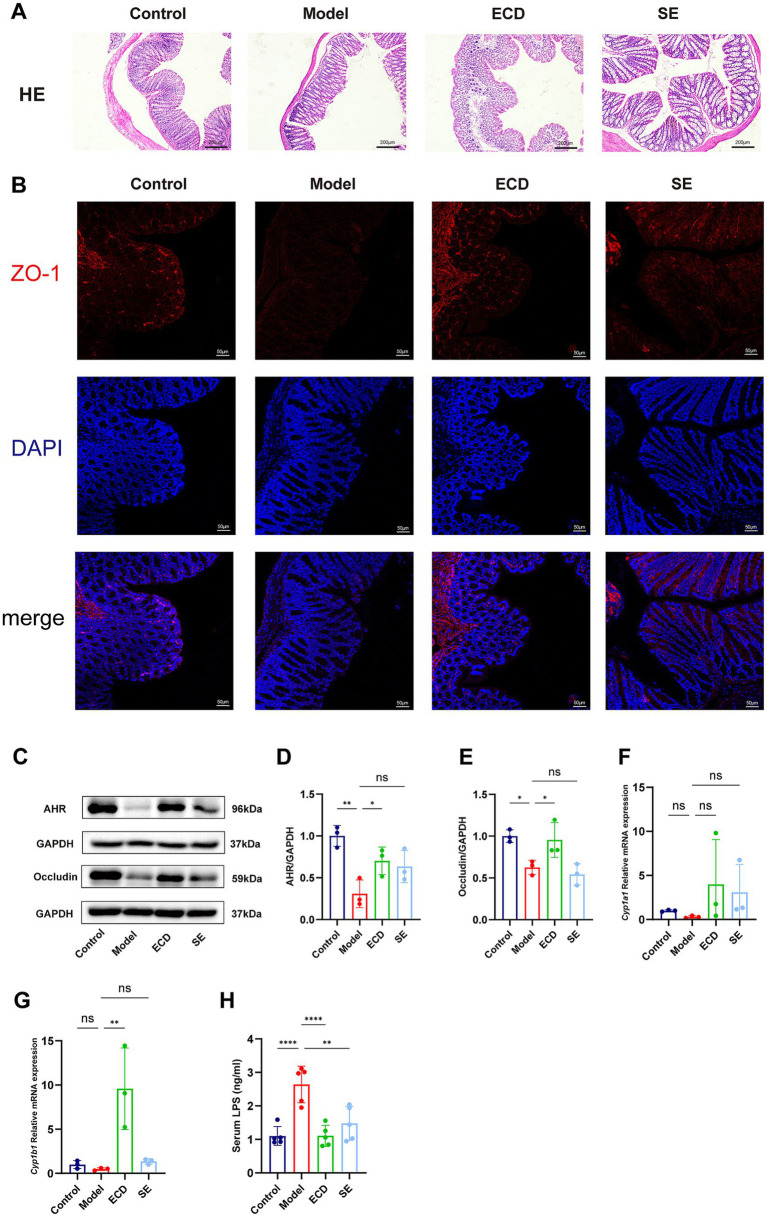
ECD activates the intestinal AHR signaling pathway to restore barrier integrity and reduce metabolic endotoxemia. **(A)** Representative H&E staining images of colonic tissues showing the general mucosal morphology (Scale bar = 200 μm). **(B)** Representative immunofluorescence images of the tight junction protein ZO-1 (red) and DAPI (blue) in colonic tissues (Scale bar = 50 μm). **(C)** Representative western blot bands of AHR and occludin proteins in colon tissue. **(D,E)** Quantitative analysis of the relative protein expression levels of AHR **(D)** and occludin **(E)**. **(F,G)** Relative mRNA expression levels of the canonical AHR target genes *Cyp1a1* **(F)** and *Cyp1b1* **(G)** in colonic tissues determined by RT-qPCR. **(H)** Serum LPS levels were determined using ELISA. Data are presented as mean ± SD (*n* = 3–5). Significance is denoted as *p < 0.05, **p < 0.01, ***p < 0.001, ****p < 0.0001, and ns, not significant.

First, we evaluated the structural integrity of the colonic barrier. H&E staining showed that the colonic mucosa in the Model group exhibited disturbed crypt architecture and reduced mucosal thickness compared to the Control group. Notably, ECD treatment improved these morphological defects, preserving the overall mucosal structure (Figure 7A).

To further visualize the tight junction assembly, we performed immunofluorescence staining for ZO-1. As shown in Figure 7B, the Control group displayed a continuous and distinct linear signal along the epithelial border. In contrast, the Model group showed a fragmented and discontinuous distribution of ZO-1. Importantly, ECD treatment restored the continuity and intensity of the ZO-1 signal. Consistent with these morphological findings, Western blot analysis confirmed that the protein expression of the tight junction protein Occludin was significantly upregulated in the ECD group compared to the Model group (Figures 7C,E).

To determine the molecular mechanism underlying this barrier restoration, we examined the AHR signaling pathway. Western blot analysis demonstrated that ECD treatment markedly increased AHR protein expression in the colon relative to the Model group (Figures 7C,D). Furthermore, to confirm the transcriptional activation of AHR, we quantified the mRNA levels of its canonical target genes. RT-qPCR results showed a significant induction of *Cyp1b1* (*p* < 0.05) and an increasing trend of *Cyp1a1* in the ECD group (Figures 7F,G). These data provide direct evidence that the elevated indoles effectively triggered AHR signaling.

Functionally, this reinforcement of the intestinal barrier effectively blocked the translocation of gut-derived endotoxins, as evidenced by the significant reduction in serum LPS levels in the ECD group (Figure 7H). These findings suggest that ECD remodels the gut microbiota to increase indole production, which subsequently activates AHR signaling to restore barrier integrity and mitigate metabolic endotoxemia, thereby reducing the influx of LPS into the liver.

### 3.7 ECD reprograms the hepatic transcriptome and reverses lipid metabolic disorders via the gut-liver axis

**Figure 8:**
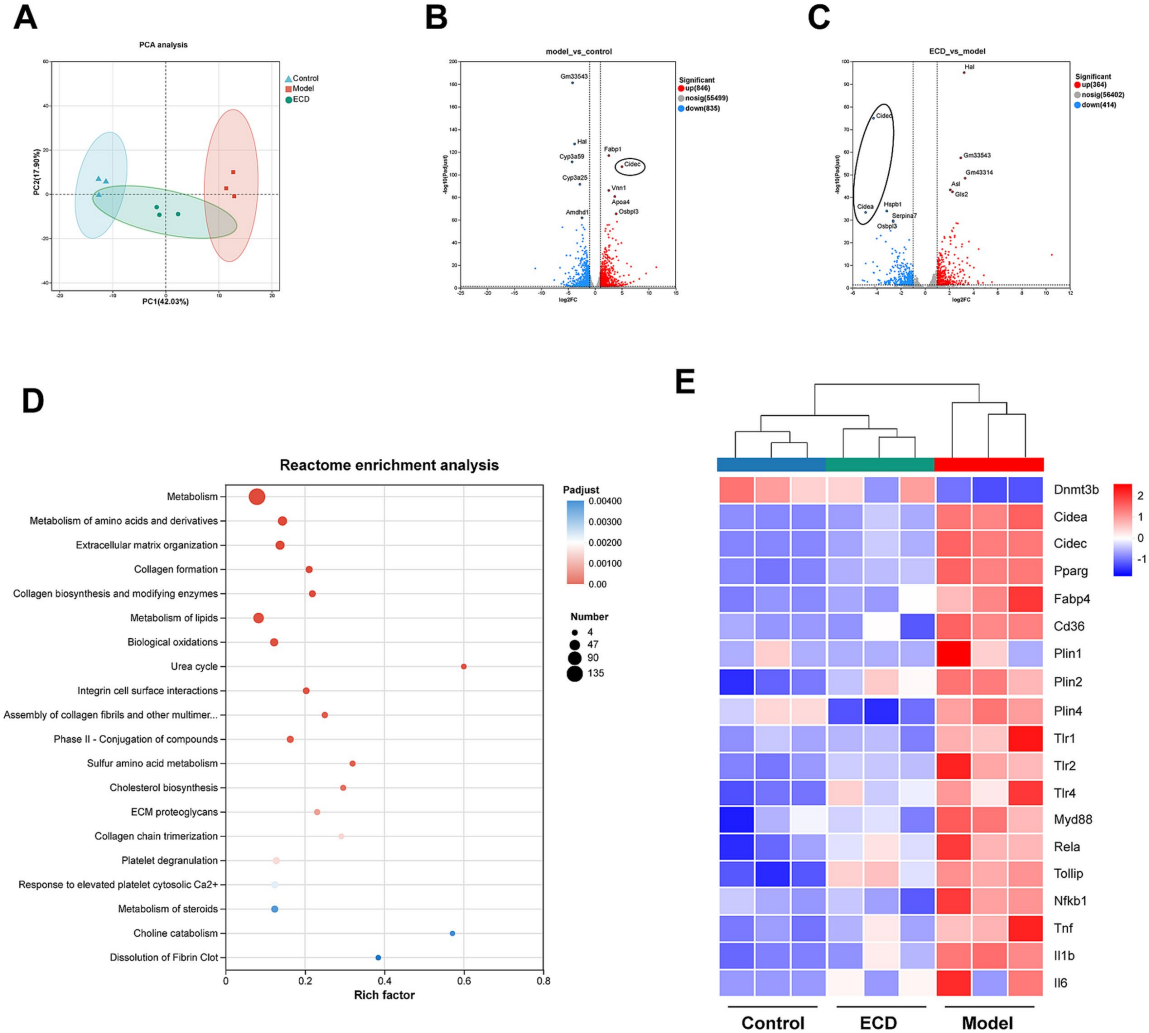
ECD reprograms the hepatic transcriptome and reverses lipid metabolic and inflammatory disorders. **(A)** Principal component analysis (PCA) plot illustrating the distinct transcriptomic separation among the control, model, and ECD groups (*n* = 3). **(B)** Volcano plot of differentially expressed genes (DEGs) between the model and control groups, highlighting the upregulated lipid droplet fusion gene *Cidec*. **(C)** Volcano plot of DEGs between the ECD and model groups, indicating the significant downregulation of *Cidec* and *Cidea*. **(D)** Reactome pathway enrichment analysis of DEGs reversed by ECD treatment. **(E)** Hierarchical clustering heatmap of key DEGs associated with lipid metabolism, inflammation, and epigenetic regulation (*Dnmt3b*). In heatmaps, red indicates high expression and blue indicates low expression. Differential analysis was conducted using DESeq2 (|log_2_FC| ≥ 1, *p* < 0.05).

Following the observation that ECD restored the intestinal barrier and reduced serum LPS levels, we investigated its downstream effects on the liver. To comprehensively explore the molecular mechanisms, we performed RNA-seq on the liver tissues. PCA analysis revealed a distinct separation among the Control, Model, and ECD groups, indicating that ECD treatment effectively shifted the hepatic transcriptomic profile away from the disease state (Figure 8A). We then examined the specific changes in gene expression that drive these profiles. As illustrated in the volcano plot comparing the Model and Control groups (Figure 8B), HFD feeding induced extensive transcriptomic alterations. Notably, the lipid droplet fusion gene, Cidec, was significantly upregulated. However, ECD treatment successfully reversed this pathological pattern. Comparison between the ECD and Model groups showed that *Cidea* and its homolog *Cidec* were significantly downregulated, indicating targeted inhibition of lipid droplet expansion (Figure 8C). To elucidate the biological functions of these changes, we performed a Reactome pathway enrichment analysis. Pathway enrichment analysis highlighted that the genes modulated by ECD were predominantly involved in metabolic processes, with a specific emphasis on lipid metabolism and biological oxidations (Figure 8D).

Furthermore, we examined the expression patterns of key genes implicated in LPS-induced inflammatory responses and lipid metabolic processes, identifying them as integral components of gut-liver crosstalk. As shown in the heatmap (Figure 8E), HFD feeding induced a broad upregulation of genes involved in LPS sensing and signaling, including *Tlr1*, *Tlr2*, *Tlr4*, *Myd88*, and *Tollip* expression. This was accompanied by elevated expression of downstream inflammatory mediators such as *Rela*, *Nfkb1*, *Tnf*, *Il1b*, and *Il6*, confirming the hepatic response to gut-derived endotoxemia. Consistent with the blockade of LPS translocation observed in Figure 7, ECD treatment systematically reversed the pathological inflammatory profile. Concurrently, genes critical for lipid droplet formation (*Cidec*, *Cidea*, *Plin1*, *Plin2*, *and Plin4*) and fatty acid uptake/synthesis (*Pparg*, *Cd36*, *and Fabp4*) were significantly downregulated. Most importantly, ECD uniquely restored the expression of *Dnmt3b*, a key epigenetic regulator suppressed by LPS.

**Figure 9:**
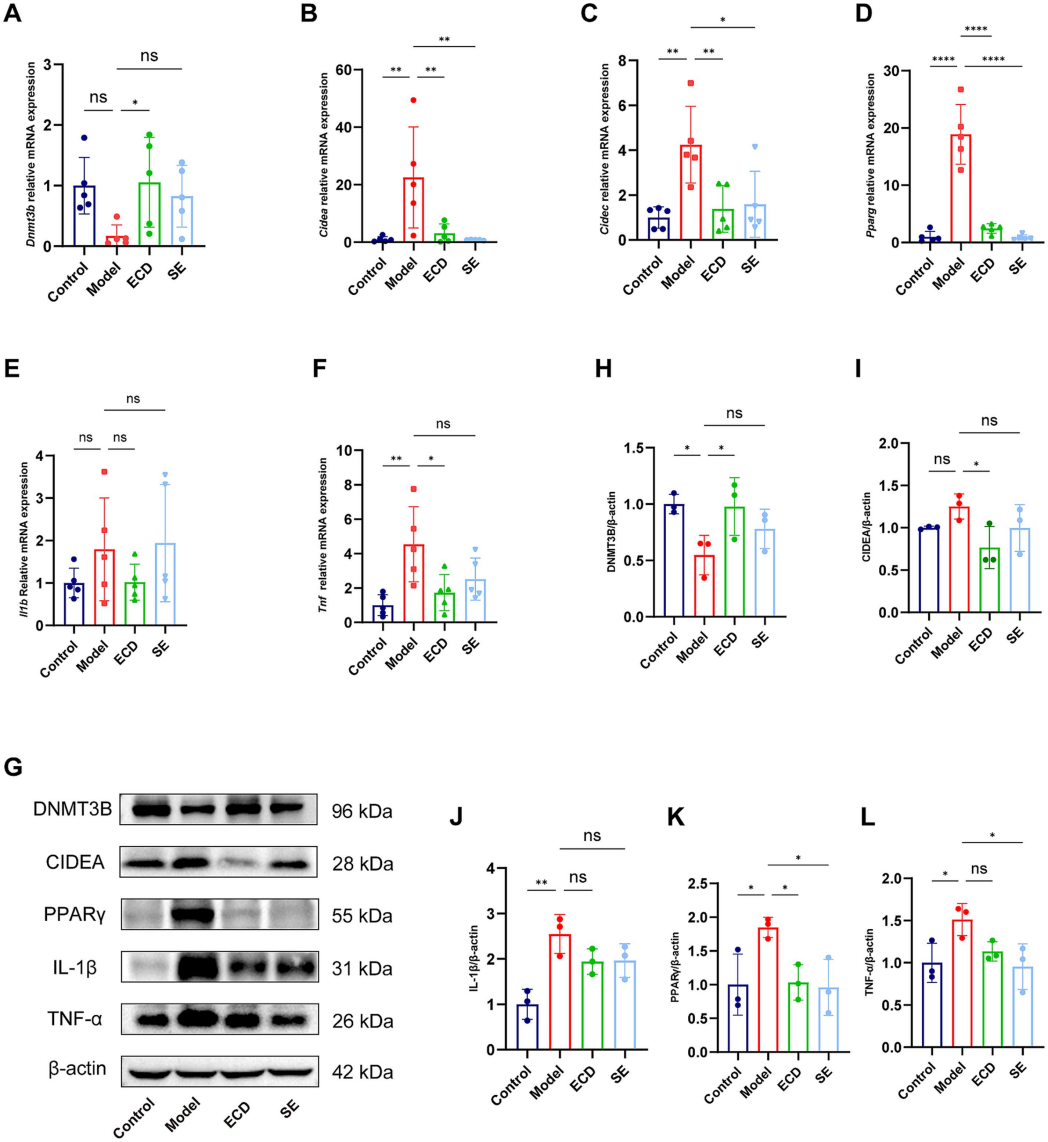
Transcriptional and translational validation of the DNMT3B-CIDEA axis and inflammatory markers in the liver. **(A–F)** Relative mRNA expression levels of *Dnmt3b* **(A)**, *Cidea* **(B)**, *Cidec* **(C)**, *Pparg* **(D)**, *Il1b* **(E)**, and *Tnf* **(F)** in liver tissues determined by RT-qPCR (*n* = 5). **(G)** Representative western blot bands of DNMT3B, CIDEA, PPARγ, IL-1β, TNF-α, and β-actin proteins in liver tissues. **(H–L)** Quantitative analysis of the relative protein expression levels of DNMT3B **(H)**, CIDEA **(I)**, IL-1β **(J)**, PPARγ **(K)**, and TNF-α **(L)** (*n* = 3). Significance is denoted as *p < 0.05, **p < 0.01, ***p < 0.001, ****p < 0.0001, and ns, not significant.

To rigorously validate these transcriptomic findings and confirm the functional execution of these gene expression changes, we performed both RT-qPCR and Western blot analyses. At the transcriptional level, RT-qPCR results showed high consistency with the RNA-seq data: the mRNA levels of *Dnmt3b* were markedly upregulated, whereas *Cidec*, *Cidea*, *Pparg*, *Tnf*, and *Il1b* were significantly downregulated in the ECD group relative to the Model group (Figures 9A–F).

Crucially, to verify these changes at the translational level, we assessed the protein abundance of key nodes in the proposed “Gut-Liver” axis. As shown in Figures 9G–L, Western blot analysis confirmed that ECD treatment significantly restored the protein expression of DNMT3B, a critical maintenance DNA methyltransferase. This restoration was accompanied by the significant suppression of the lipid droplet fusion protein CIDEA and the key lipogenic transcription factor PPARγ. Furthermore, consistent with the alleviated hepatic inflammation, the protein levels of pro-inflammatory cytokines TNF-α were reduced, while IL-1β exhibited a consistent downward trend. Collectively, these multi-level validations demonstrate that ECD ameliorates MASLD by attenuating LPS-driven hepatic inflammatory and metabolic responses, potentially mediated by the DNMT3B-dependent epigenetic remodeling of the hepatic landscape.

## 4 Discussion

In this study, we systematically investigated the therapeutic effects and underlying mechanisms of ECD in HFD-induced MASLD mice using an integrated multiomics strategy. Our results demonstrated that ECD significantly alleviated hepatic steatosis, insulin resistance, and dyslipidemia, exhibiting therapeutic efficacy comparable to that of SE.

Gut microbiota dysbiosis is a key driver of MASLD progression, and targeted modulation of the microbiome is a promising therapeutic strategy (Bahitham et al., 2025; Borges-Canha et al., 2025; Chen and Vitetta, 2020). Extensive evidence indicates that chronic HFD feeding disrupts intestinal microbial homeostasis, characterized by reduced diversity and structural shifts, which exacerbates metabolic disorders (Daniel et al., 2014; Lau et al., 2025). In our study, both ECD and SE treatments ameliorated HFD-induced dysbiosis, shifting the overall microbial community structure toward a healthy profile. Notably, our study revealed distinct regulatory patterns between ECD and SE. Consistent with previous reports and our metagenomic analysis, the gut microbiota modulation by SE was characterized by the enrichment of *Akkermansia* (Duan et al., 2024; Feng et al., 2024; Sun et al., 2025). *Akkermansia,* a mucin-degrading bacterium known to improve metabolic health, has been reported to enhance lipid oxidation and bile acid metabolism, maintain gut barrier integrity, and reshape gut microbiota composition, thereby suppressing hepatic inflammation and ameliorating MASLD (Han et al., 2023; Rao et al., 2021). In contrast, ECD uniquely enriched *the Lactobacillus* and *Bifidobacterium* populations. These bacteria are known to promote the production of indole derivatives (e.g., Indole-3-Acetic Acid and indoleacrylic acid) (Zelante et al., 2013; Zhang et al., 2025). Our serum metabolomics analysis demonstrated significant systemic accumulation of indole derivatives in the ECD group. This suggests that ECD likely exerts hepatoprotective effects through a distinct “gut microbiota-indole-barrier” axis.

Microbiota-derived indole derivatives act as natural ligands for AHR (Wlodarska et al., 2017). AHR is a ligand-activated transcription factor that serves as a critical sensor of the intestinal environment and guardian of mucosal integrity (Pinto et al., 2023). Upon binding to these microbiota-derived ligands, AHR is activated and translocates to the nucleus to transcriptionally regulate target genes involved in epithelial renewal, tight junction assembly, and anti-inflammatory responses (Chng et al., 2016; Pinto et al., 2023; Yu et al., 2018). Consistent with this, we observed that ECD treatment significantly upregulated colonic AHR expression. Importantly, this activation was accompanied by the comprehensive restoration of intestinal barrier integrity, as evidenced by the improved colonic morphology (H&E staining) and the upregulated expression of tight junction proteins ZO-1 and Occludin.

Physiologically, the intestinal barrier restricts the translocation of gut-derived endotoxins (LPS) into the systemic circulation (Ghorbani et al., 2025; Ghosh et al., 2020). However, chronic HFD feeding disrupts this barrier integrity, leading to leaky gut and subsequent metabolic endotoxemia (Miranda et al., 2024; Rohr et al., 2020), as evidenced by the suppressed Occludin expression and markedly elevated serum LPS levels in the Model group. Importantly, our results showed that ECD treatment effectively reversed HFD-induced pathological changes, restored barrier function, and significantly reduced serum LPS levels. Collectively, these findings indicate that ECD ameliorates HFD-induced intestinal barrier dysfunction and mitigates metabolic endotoxemia, at least in part, via AHR signaling activation.

The gut-liver axis is a critical regulator of hepatic lipid metabolism. Previous studies have extensively explored the therapeutic mechanisms of ECD in MASLD from various perspectives. For instance, Wang et al. demonstrated that ECD and its active compounds ameliorate MASLD by activating the AMPK signaling pathway to regulate lipid metabolism (Wang Y. et al., 2025). Deng et al. revealed a novel mechanism by which ECD inhibits lipid accumulation and iron overload via the Caveolin-1 signaling pathway (Deng et al., 2024). Additionally, Miao et al. highlighted the role of ECD in remodeling the gut microbiota and regulating serum metabolism, involving taurine and sulfur amino acid pathways (Miao et al., 2022). Recent integrative studies have further summarized that ECD exerts hepatoprotective effects through multiple targets, including inflammation and oxidative stress regulation (Zheng et al., 2025). However, despite these advances, the potential epigenetic mechanisms linking gut-derived signals to hepatic metabolic reprogramming remain largely uninvestigated. In this study, we verified that a HFD resulted in a significant elevation of serum lipopolysaccharide (LPS) levels, indicating a pathological translocation of gut-derived endotoxins into the liver. This influx was effectively mitigated by ECD treatment. To unravel the downstream hepatic response to the blockade of endotoxin influx, we performed hepatic transcriptomic sequencing, which revealed that sequencing revealed that ECD treatment induced a distinct shift in gene expression profiles, characterized by the broad suppression of inflammatory mediators and key lipogenic regulators. These findings were validated by both RT-qPCR and Western Blot assays, which confirmed that ECD downregulated the expression of inflammatory cytokines (TNF-α, IL-1β) and the key lipogenic regulator (PPARγ) at both the transcriptional and translational levels.

Building on the regulation of classical pathways, we specifically focused on the novel LPS-DNMT3B-CIDEA axis. Recent pivotal research has established that chronic low-grade LPS functions not merely as an inflammatory trigger but as an epigenetic modifier: LPS-induced suppression of DNMT3B leads to the hypomethylation and transcriptional activation of Cidea, thereby driving lipid droplet fusion and macrovesicular steatosis (Li Q. et al., 2024). Consistent with this epigenetic mechanism, we found that the expression of Dnmt3b was significantly suppressed, whereas the lipid droplet fusion gene Cidea/Cidec was markedly upregulated in the Model group. Importantly, ECD treatment effectively reversed this pathological pattern, restoring Dnmt3b levels and suppressing Cidea expression at both the transcriptional (RT-qPCR) and translational (Western Blot) levels. Collectively, these results suggest that complementing its regulation of classical lipid metabolic pathways, ECD exerts its therapeutic effects through an epigenetic dimension. Specifically, by mitigating the LPS-induced suppression of Dnmt3b and subsequent Cidea activation, ECD prevents pathological lipid droplet expansion.

In conclusion, our results indicate that ECD may alleviate hepatic steatosis by orchestrating the gut microbiota-indole-AHR axis to repair the intestinal barrier and block pathogenic LPS influx, thereby triggering DNMT3B-mediated hepatic epigenetic reprogramming to suppress CIDEA expression and inhibit lipid droplet expansion.

Despite these promising findings, our study has certain limitations. First, although we confirmed the involvement of the Dnmt3b-Cidea pathway at both transcriptional and translational levels, direct detection of Cidea promoter methylation levels (e.g., via Bisulfite Sequencing PCR) would provide more definitive evidence of this epigenetic regulation, which we aim to address in future investigations. Second, regarding metabolomics, while we identified key indole derivatives, their absolute concentrations were not quantified by targeted metabolomics or HPLC due to sample limitations; however, the functional activation of AHR target genes provides indirect support for their enrichment. Third, while we evaluated intestinal barrier function through molecular markers (AHR, ZO-1, and Occludin) and histological assessments (H&E staining), more advanced morphological observations, such as transmission electron microscopy, could further elucidate the ultrastructural changes. Fourth, our study was conducted exclusively on male mice to minimize the impact of hormonal fluctuations on metabolic phenotypes. Future research incorporating female mice is warranted to determine whether these therapeutic effects and the identified ‘gut-liver’ mechanisms are consistent across sexes. Fifth, while the current study demonstrated efficacy over the treatment period, the long-term durability of these effects after the cessation of ECD remains unknown due to the lack of a wash-out observation period. Additionally, although we confirmed hepatoprotection (reduced ALT/AST) and observed no overt signs of systemic toxicity, comprehensive toxicological assessments, such as renal function panels and histopathology of non-target organs (e.g., heart and kidney), were not performed. Future studies should include these evaluations to fully establish the preclinical safety profile. Finally, the causal relationship between specific bacteria (Lactobacillus and Bifidobacterium) and indole production was established based on correlation and literature; further validation using germ-free mice or fecal microbiota transplantation, combined with isotope-labeled tryptophan tracing to track metabolic flux, is required to definitively confirm the microbial contribution and the direct conversion of ECD components.

## Data Availability

The raw sequencing data generated in this study have been deposited in the NCBI Sequence Read Archive (SRA). The fecal metagenomic data are available under BioProject accession number PRJNA1380942, and the liver transcriptomic data are available under BioProject accession number PRJNA1380962.

## Footnotes

1
https://cloud.majorbio.com

## Glossary

AHR: Aryl hydrocarbon receptor
ALT: Alanine aminotransferase
AST: Aspartate aminotransferase
CIDEA: Cell death-inducing DFFA-like effector a
CIDEC: Cell death-inducing DFFA-like effector c
DNMT3B: DNA methyltransferase 3B
ECD: Er-Chen Decoction
FDR: False discovery rate
HFD: High-fat diet
LDA: Linear discriminant analysis
LEfSe: Linear discriminant analysis effect size
LPS: Lipopolysaccharides
MASLD: Metabolic dysfunction-associated steatotic liver disease
MASH: Metabolic dysfunction-associated steatohepatitis
NAFLD: Non-alcoholic fatty liver disease
OGTT: Oral glucose tolerance test
PCA: Principal component analysis
PCoA: Principal coordinate analysis
PLS-DA: Partial least squares discriminant analysis
SE: Semaglutide
T2DM: Type 2 diabetes mellitus
TC: Total cholesterol
TCM: Traditional Chinese medicine
TG: Triglycerides
TLR4: Toll-like receptor 4
TPM: Transcripts per million
UPLC-MS/MS: Ultra-performance liquid chromatography–tandem mass spectrometry
