# Supplementary material for: Er-Chen Decoction ameliorates metabolic dysfunction–associated steatotic liver disease via gut microbiota-barrier axis-driven hepatic metabolic reprogramming
Source: Front Microbiol. 2026 Mar 10;17:1768664. doi: 10.3389/fmicb.2026.1768664 (PMC13033753; doi:10.3389/fmicb.2026.1768664)
Supplement: Supplementary file 1 [file Supplementary_file_1.docx]

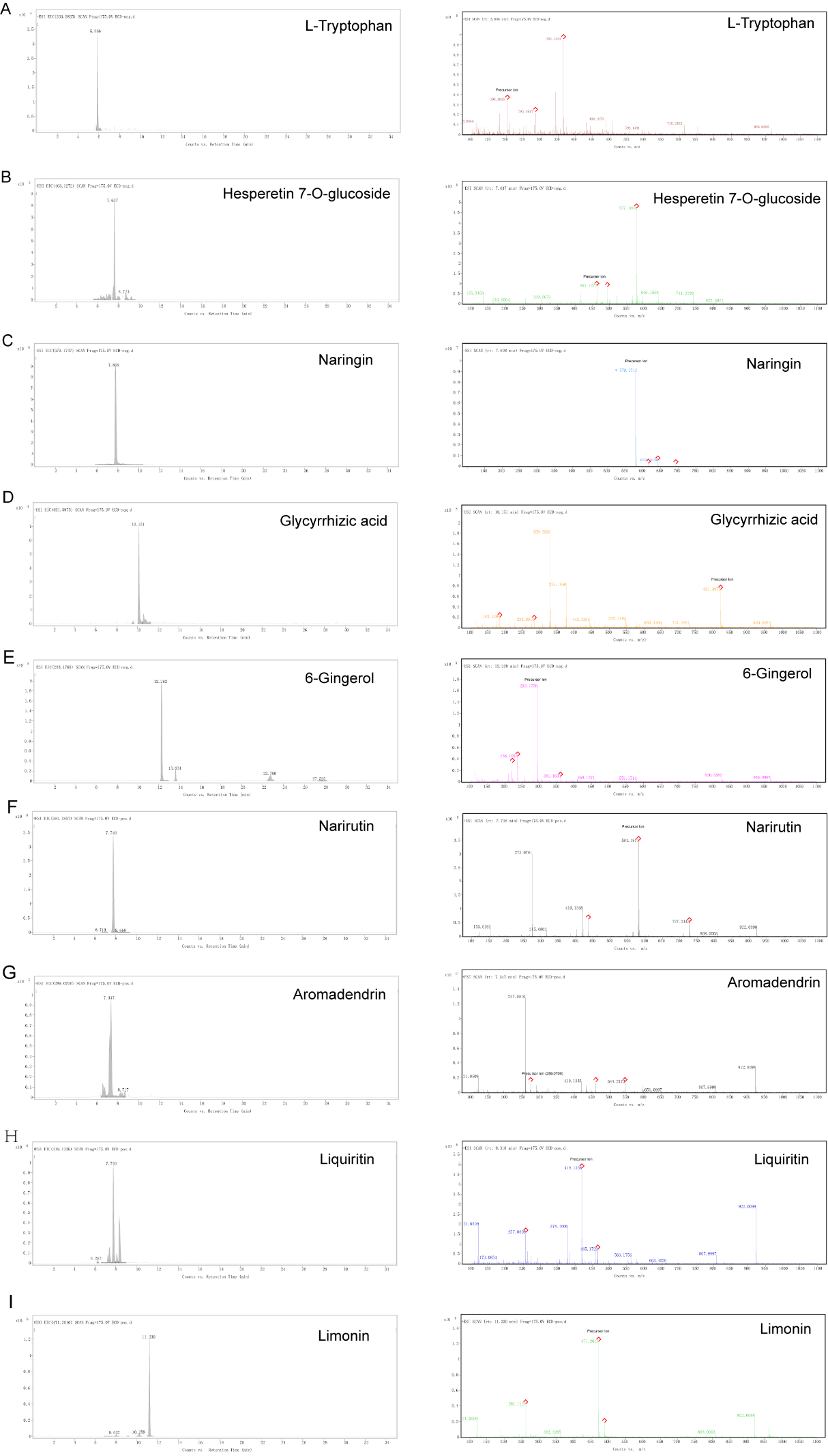


**Supplementary Figure S1. Extracted ion chromatograms (EICs) and MS spectra of the nine representative chemical constituents in ECD.**

The figure displays the EIC and corresponding MS/MS fragmentation spectra for each of the nine key compounds identified in Er-Chen Decoction. These compounds correspond to the retention times indicated in Figure 1 and are listed as follows: (A) L-Tryptophan, (B) Hesperetin 7-O-glucoside, (C) Naringin, (D) Glycyrrhizic acid, (E) 6-Gingerol, (F) Narirutin, (G) Aromadendrin, (H) Liquiritin, and (I) Limonin.


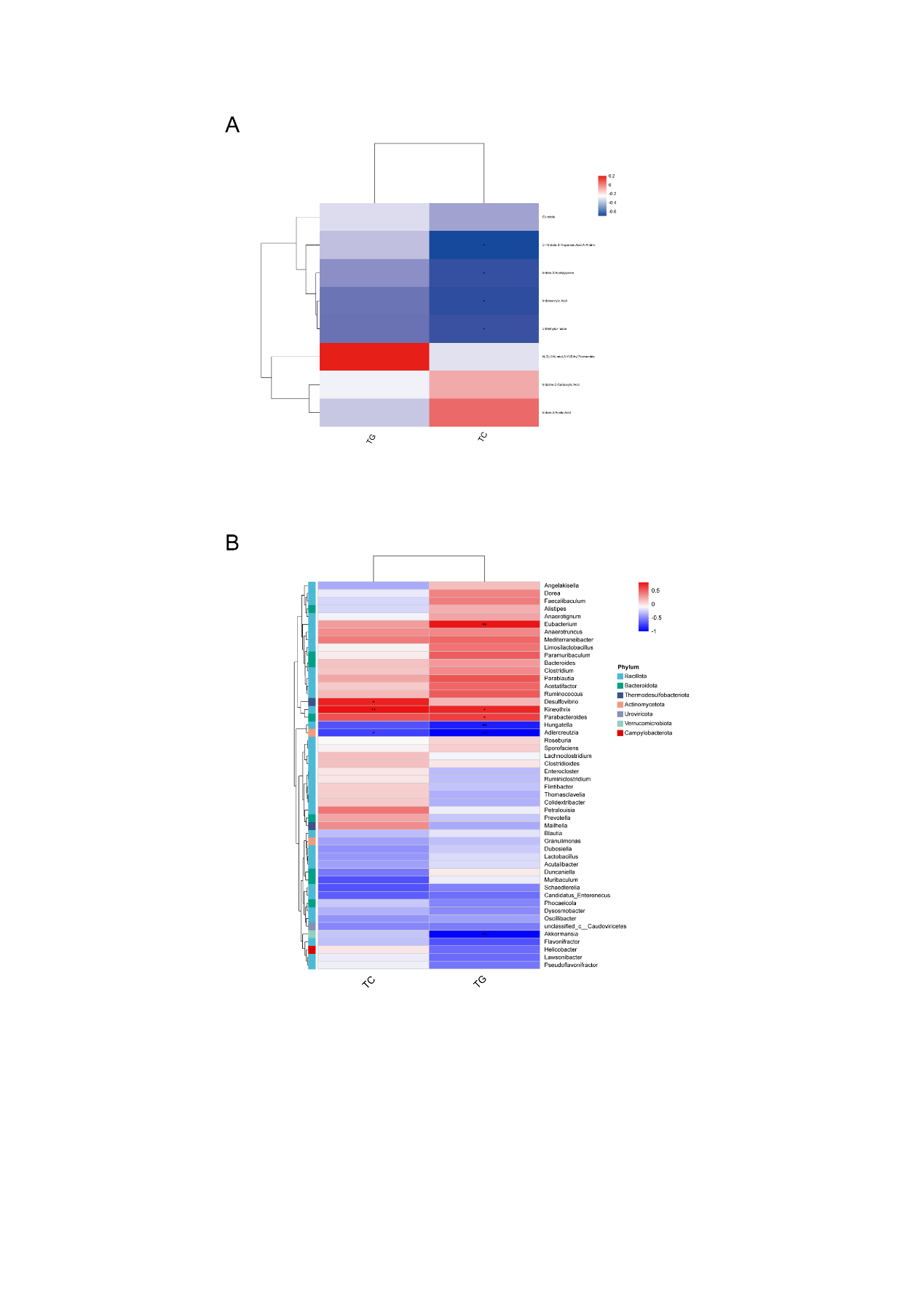


**Supplementary Figure S2. Spearman correlation analysis linking the gut microbiome and metabolome to host lipid phenotypes.**

(A) Heatmap showing the Spearman correlation coefficients between differential serum metabolites (specifically AHR-activating indole derivatives) and lipid parameters (TG and TC). (B) Heatmap showing the Spearman correlation coefficients between differential gut bacterial genera and lipid parameters. The color scale indicates the correlation strength, where red represents a positive correlation and blue represents a negative correlation. Statistical significance is indicated by asterisks (*P < 0.05, **P < 0.01).
